# Spontaneous Retroperitoneal Hematoma in SARS-CoV-2 Patients: Diagnostic and Management Challenges—A Literature Review

**DOI:** 10.3390/jcm14196999

**Published:** 2025-10-03

**Authors:** Alexandra Sandu, Dan Bratu, Alin Mihețiu, Dragos Serban, Ciprian Tănăsescu

**Affiliations:** 1 Department of Surgery, County Clinical Emergency Hospital of Sibiu, 550245 Sibiu, Romania; alexandrae.sandu@ulbsibiu.ro (A.S.); alin.mihetiu@ulbsibiu.ro (A.M.); ciprian.tanasescu@ulbsibiu.ro (C.T.); 2 Faculty of Medicine, Lucian Blaga University of Sibiu, 550169 Sibiu, Romania; 3 Department of Surgery, Carol Davila University of Medicine and Pharmacy, 020021 Bucharest, Romania; dragos.serban@umfcd.ro

**Keywords:** SARS-CoV-2, spontaneous retroperitoneal hematoma, anticoagulant therapy, spontaneous hemorrhages

## Abstract

**Background:** Spontaneous retroperitoneal hematomas constitute a rare clinical entity, yet their incidence has markedly increased during the SARS-CoV-2 pandemic. The pathophysiological substrate is incompletely elucidated, being influenced by anticoagulant therapy, vascular inflammatory alterations induced by SARS-CoV-2 infection, and comorbidities in critically ill patients that exacerbate hemorrhagic risk. **Methods:** We performed a comprehensive literature review of published case reports and case series on spontaneous retroperitoneal hematomas in COVID-19 patients, complemented by our institutional experience, in order to synthesize current diagnostic and therapeutic approaches. **Results:** Available evidence indicates that most cases occur in anticoagulated patients, with clinical manifestations often limited to nonspecific abdominal or lumbar pain. Diagnosis relies primarily on contrast-enhanced CT imaging. Reported therapeutic strategies include conservative management, endovascular embolization, and surgical intervention, with outcomes ranging from complete recovery to fatal progression, particularly in elderly and comorbid individuals. **Conclusions:** Spontaneous retroperitoneal hematomas in the setting of SARS-CoV-2 infection represent a diagnostic and therapeutic challenge associated with considerable morbidity and mortality. Early recognition, prompt imaging, and individualized multidisciplinary management are essential, while further research is needed to clarify incidence, risk factors, and preventive strategies.

## 1. Introduction

The first case of SARS-CoV-2 infection was reported by the World Health Organization in December 2019. As the disease spread and became a global pandemic, an increased incidence of embolic events in these patients was observed, leading to the addition of anticoagulant therapy. While mortality rates have improved, a significant rise in spontaneous hemorrhages has also been noted. The incidence of spontaneous hemorrhages in COVID-19 patients ranges from 2 to 5%, depending on the study analyzed, and increases to 7–8% in critically ill patients [1].

Retroperitoneal hemorrhages and hematomas are primarily caused by local trauma or secondary bleeding from retroperitoneal tumors [2].

Spontaneous retroperitoneal hematoma, prior to the onset of the pandemic, was a rarely encountered condition, primarily described in the context of Wunderlich syndrome. Causes of retroperitoneal hemorrhage can include ruptures of angiomyolipomas, renal cysts, or ruptures of aneurysms in renal, lumbar, or even gluteal vessels. Another possible cause is prolonged and high-dose anticoagulant therapy, particularly in elderly patients, who show a higher incidence of this condition. However, 15% of those who developed spontaneous retroperitoneal hematomas had no association with anticoagulant or antiplatelet therapy. The clinical manifestations are nonspecific, with progression sometimes insidious until hematoma rupture occurs into the peritoneal cavity, leading to signs of hemorrhagic shock. Symptoms typically include lower back pain radiating to the buttocks and lower limb, the appearance of lumbar or abdominal ecchymosis, and systemic manifestations of anemia or hemorrhagic shock. Rupture into the abdominal cavity is associated with acute surgical abdomen due to hemorrhagic retroperitoneum [3].

The nonspecific manifestations in patients frequently in intensive care, or sometimes even intubated; the rarity of this pathology; the recent connection between its increased incidence in COVID-19 patients; as well as the therapeutic dilemmas and often unfavorable outcomes necessitate an analysis aimed at guiding more effective diagnostic and therapeutic measures.

## 2. Results

### 2.1. Materials and Methods

**Figure 1:**
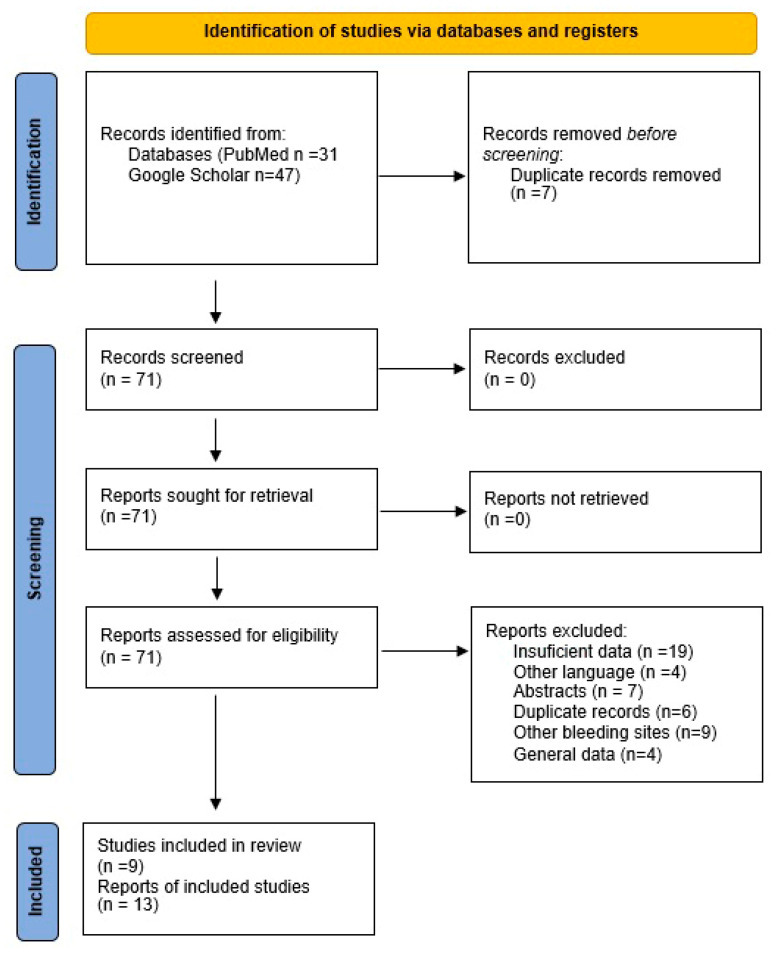
PRISMA (Preferred Reporting Items for Systematic Reviews and Meta-Analyses) flow chart on the literature review regarding the occurrence of spontaneous retroperitoneal hematomas in patients with SARS-CoV2.

We conducted a review of the PubMed and Google Scholar databases by entering the keywords “spontaneous retroperitoneal hematoma” and “COVID-19/SARS-CoV-2” with a timeline from 2020 to 2024. We included original studies, case reports, and case series that documented spontaneous retroperitoneal hematoma in adult patients with confirmed SARS-CoV-2 infection. Eligible articles were required to provide adequate clinical and/or imaging information to support the diagnosis and to allow extraction of relevant data regarding patient management and outcomes. Exclusion criteria comprised non-original studies (reviews, editorials, conference abstracts), non-English publications, reports lacking clinical or imaging data, pediatric cases, and studies unrelated to retroperitoneal hematoma in SARS-CoV-2 patients. The PubMed search yielded 31 results, from which 13 articles were selected after applying exclusion criteria, including 4 case series and 9 case reports, identifying a total of 30 patients. The Google Scholar search identified 47 results, and after applying exclusion criteria, 9 articles were analyzed, including 24 patients—4 as case reports and 5 as case series. The results were synthesized using a Prisma flow chart Figure 1 and Table 1. In order to ensure methodological rigor and transparency, this literature review was structured following the PRISMA 2020 guidelines. Nevertheless, the review protocol was not registered in an international registry (e.g., PROSPERO), as the scope was primarily to synthesize published evidence and integrate it with our clinical experience. Incomplete reporting of vaccination status in the included studies limited our ability to analyze its potential impact on the development and outcomes of retroperitoneal hematoma in COVID-19 patients.

This type of analysis may be subject to a risk of bias regarding the degree of representativeness of the patients, given that the majority of cases with this type of complication arise from critically ill patients. Additionally, the degree of heterogeneity among the published studies on this subject may introduce bias due to the inclusion criteria and the types of data collected in the studies. The recent nature of this condition may impact the results of the analysis due to the absence of a sufficiently long follow-up. Last but not least, the pandemic nature of the viral infection with COVID-19, characterized by peaks of infection and the progression toward stabilization through immunization, directly affects the achievement of heterogeneous results concerning the identification or reporting of retroperitoneal hematomas.

To minimize these risks regarding bias, two authors independently selected the cases included in the study; the agreement ratio for included studies was 95%, while it was 97% for excluded articles. The collected data were organized using Microsoft Excel 2019 (Microsoft Corp., Redmond, WA, USA) for statistical analysis, utilizing SPSS Data Analysis Software 28.0.1 and DATAtab Team (2024). DATAtab: Online Statistics Calculator. DATAtab e.U., Graz, Austria. Descriptive statistics were used to summarize patient demographics, clinical characteristics, and outcomes. Categorical variables were expressed as frequencies and percentages, while continuous variables were reported as mean ± standard deviation. Comparative analyses between groups were performed using the chi-square test or Fisher’s exact test for categorical variables and Student’s *t*-test for continuous variables. A *p*-value < 0.05 was considered statistically significant.

Demographic data and significant clinical, imaging, and biological data prior to diagnosis, as well as therapeutic management and outcome data, were evaluated.

### 2.2. Results

#### 2.2.1. General Patient Data, Clinical, Imaging, and Biological Characteristics

**Figure 2:**
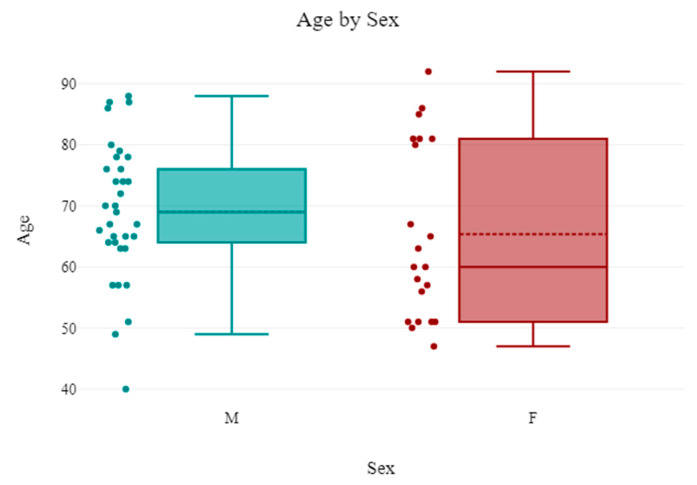
Diagram representing the association between age and gender of patients with spontaneous retroperitoneal hematoma and SARS-CoV-2 infection.

The average age identified was 67.61 ± 12.56 years, with a higher prevalence of male patients at 61.11% (*n* = 33). The average age for male patients was 69.03 ± 11.25 years, while for female patients it was 65.38 ± 14.4 years (Figure 2).

Analysis by year indicated that 13.63% of cases occurred in 2020, 45.45% in 2021, 27.27% in 2022, and 13.63% in 2023. The mean time from initial diagnosis to the detection of retroperitoneal hematoma was 11.19 ± 8.05 days, with the minimum interval from SARS-CoV-2 infection to the development of retroperitoneal hemorrhage being 2 days.

Of the 53 patients included in the study, 4 (7.54%) experienced respiratory deterioration that necessitated transfer to the intensive care unit and initiation of mechanical ventilation. These events were identified in the context of pronounced hemodynamic instability and anemia.

The most commonly reported symptom was back pain (38.89%), followed by diffuse abdominal pain, with the right retroperitoneal space being the predominant site (57.41%), as illustrated in Table 2.

A statistically significant relationship between the type of clinical manifestation and the location of the hematoma was observed, indicated by *p* values < 0.001 (Chi-Square test).

The regression statistical analysis highlighted the relationship between specific types of symptoms and the tendency toward a particular location of the hematoma collection, with statistically significant results presented in the following table—Table 3.

The average size of the collections was 16.31 ± 6.36 cm, with a maximum of 27 cm. The progression to larger sizes was more frequent in the left retroperitoneal space; however, this difference did not reach statistical significance (*p* > 0.05).

The most commonly used anticoagulant therapy was Enoxaparin, particularly in the form of 0.4 IU (25.93%) as a single dose, as well as variations of 0.4 × 2, 0.6, or 0.6 × 2.

Analyzing a possible correlation between the type of anticoagulant therapy and the duration from initiation to the occurrence of retroperitoneal hematomas, it was observed that for Enoxaparin 0.4, the duration was 12.21 ± 9.74 days, while for the group receiving Heparin 5000 IU, it was 7.13 ± 3.6 days. The administration of Warfarin or Heparin 10,000 IU registered a nonsignificant number of patients experiencing hemorrhagic phenomena 3 days after the initiation of therapy.

Although variations were noted between the association of a specific type of anticoagulant therapy and the duration until the onset of retroperitoneal bleeding, the statistical analysis did not reveal a correlation between these parameters (*p* > 0.05).

At the time of diagnosis with retroperitoneal collection, the mean value of hemoglobin (Hgb) was 8.97 ± 2.63 g/dL. Collections located on the left side were also associated with more severe anemia compared to those on the right retroperitoneal side, with values of 8.02 ± 2.15 g/dL versus 9.65 ± 2.8 g/dL, though this difference did not achieve statistical significance (*p* = 0.056).

#### 2.2.2. Interventional Data and Progression

In 55.56% of cases (n = 30), the preferred therapeutic management was conservative. An equal percentage (22.22%) of patients underwent either embolization or surgical intervention, which consisted of hematoma evacuation, local hemostasis, and management. The therapeutic options based on the location of the collection are presented in Table 4.

The lower average hemoglobin (Hgb) values somewhat paradoxically resulted in a conservative approach toward the retroperitoneal collection. However, the difference between the chosen therapeutic solution and the degree of anemia did not reach statistical significance (*p* > 0.05). This observation also applies to the analysis of the correlation between the type of anticoagulant used and the preference for one of the therapeutic options (Table 5).

Significantly low Hgb values were associated with a higher mortality rate, demonstrating a statistically significant correlation between these two variables (*p* = 0.003).

**Figure 3:**
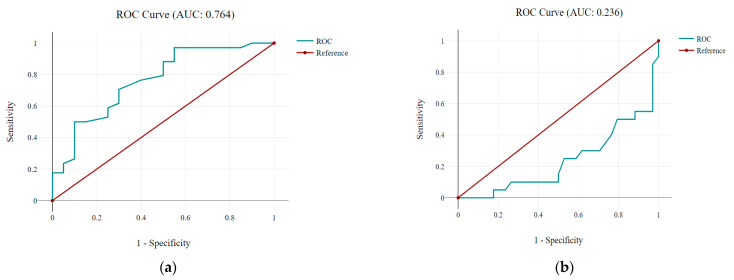
ROC analysis: correlation between Hgb levels and patient outcomes. (**a**) indicates a good discriminative ability, suggesting that higher hemoglobin values are associated with favorable outcomes; (**b**) demonstrates the predictive performance of hemoglobin levels for patient outcomes.

The ROC (Receiver Operating Characteristic) analysis highlights the relationship between higher hemoglobin values and favorable patient outcomes, with an AUC of 0.764 (Figure 3).

Both surgical and embolization approaches were associated with a 75% rate of favorable outcomes, whereas conservative management, although more frequently employed, was linked to a survival rate of 53.33%. Analysis of the potential association between management strategy and clinical outcome demonstrated differences across the three treatment modalities; however, these did not reach statistical significance (*p* > 0.05). It is also important to emphasize that conservative management was primarily chosen for patients in critical condition, in whom interventional procedures were contraindicated, a circumstance that likely influenced the observed survival rates in this group (Table 6).

## 3. Discussion

SARS-CoV-2 infection primarily manifests as lung infection and impairment, leading to inflammatory phenomena associated with pneumonia, acute respiratory distress syndrome, or pulmonary damage characterized by the cytokine storm in certain patient categories.

A unique aspect of this pathogen is its progression toward a pro-coagulant state, which can lead to thrombotic events ranging from microthrombi to large vessel thromboses. The pathophysiological mechanism underlying thrombosis in COVID-19 infections remains incompletely understood; it appears to result from a combination of factors rather than a single cause. Key contributing factors include endothelial wall damage due to inflammation caused by viral infection of endothelial cells. These dysfunctional cells release interleukins (IL-6 and IL-1β), which exacerbate local inflammation, reduce vascular lumen, and increase blood viscosity [24,25].

In a paradoxical manner, patients with thrombocytopenia during COVID-19 infection can develop thrombosis, which can be explained by the hyperreactivity of residual platelets. Alterations in the renin–angiotensin–aldosterone system, progression toward disseminated intravascular coagulation (DIC), prolonged immobilization—especially in critically ill patients—and genetic predisposition are all factors that contribute to thrombotic coagulopathies associated with this type of infection [26,27].

As a countermeasure against these complications, anticoagulant therapy has been implemented, sometimes at doses exceeding usual levels. This approach has reduced the number of cases progressing to severity, improved survival rates, and decreased thrombotic events but has also been associated with spontaneous hemorrhagic phenomena of varying degrees and occasionally unexpected locations. Musoke et al. report in a retrospective analysis that 11% of patients with COVID-19 receiving therapeutic doses of anticoagulants experienced bleeding, compared to 4% in those on prophylactic doses, with 11% of these patients developing severe hemorrhagic forms and a mortality rate of 40% for cases of severe bleeding [28].

The most common sites of bleeding in patients with SARS-CoV-2 infection have been gastrointestinal, hemorrhagic strokes, and other locations, particularly the abdominal wall, peritoneal cavity, and, less frequently, the retroperitoneum. Gastrointestinal hemorrhages in COVID-19 patients have an incidence ranging from 3% to 13%, with the stomach and small intestine being the predominant sources of bleeding [29].

The presence of angiotensin-converting enzyme 2 (ACE2) receptors in the intestines allows for the virus to penetrate enterocytes, triggering inflammatory mechanisms, cellular destruction, ischemia, and vascular thrombosis, typically resulting in mucosal and submucosal hemorrhages. These can progress to extensive diffuse forms, necessitating resections or vascular exclusions [29].

Intraperitoneal and parietal hemorrhages in patients with COVID-19 are primarily caused by the pathophysiological mechanisms of vascular wall destruction due to inflammatory processes, partial vascular ischemia, vascular breaches, or complications associated with anticoagulant treatment [30].

Spontaneous retroperitoneal hematomas are a rare entity, more frequently associated with Wunderlich syndrome (potentially life-threatening spontaneous renal hemorrhage into subcapsular and perirenal spaces). Their occurrence in patients with COVID-19 has led to an increase in reported cases of retroperitoneal bleeding. The mechanisms are debatable, with inflammatory–ischemic processes implicated in the context of direct viral damage or therapeutic anticoagulation leading to diffuse retroperitoneal bleeding or bleeding from lobar vascular elements [31].

While manifestations in other locations may alert the medical team, the symptoms of retroperitoneal hematomas are nonspecific. The most common symptoms include diffuse abdominal pain, lumbar pain, or pain radiating to the lower limb, which is usually not intense and may be tolerable in the initial phase. The analysis conducted reveals a diverse range of symptoms, which a critically ill patient may not be able to articulate, making the diagnosis even more uncertain and sometimes delayed [31].

The association of pain phenomena, hemodynamic instability, and sudden-onset anemia without signs of extravasation should raise suspicion of intra-abdominal or retroperitoneal blood loss. Analyzing the characteristics of intraperitoneal hemorrhages in comparison to retroperitoneal ones reveals significant anatomical differences. Retroperitoneal hemorrhages evolve more slowly, with less alarming pain symptoms and less abrupt biological and hemodynamic changes than intraperitoneal hemorrhages. However, clinical and biological pictures can rapidly deteriorate when retroperitoneal collections reach large sizes, leading to quick deterioration through large-diameter vascular sources or through retroperitoneal dissection and breach of the posterior parietal peritoneum, resulting in rupture into the abdominal cavity [31,32].

The average duration from the diagnosis of this type of infection to the identification of retroperitoneal collection suggests the existence of a complex and prolonged mechanism leading to vascular breach, likely due to viral inflammatory damage to the vascular endothelium or prolonged anticoagulant therapy, rather than an acute, purely vascular mechanism. Our analysis highlights this pattern, noting that, on average, the diagnosis of bleeding occurred 11 days after confirming COVID-19 infection [33].

Regarding the type of anticoagulant used, there is a preference for low molecular weight heparins, such as Enoxaparin, over warfarin; however, no statistically significant importance was detected regarding a potential correlation between anticoagulant therapy and the size of the hematomas or the timing of hematoma diagnosis in relation to the timing of the infection. When comparing therapeutic doses with prophylactic doses, especially with high doses of Enoxaparin (1 g, *p* = 0.021), we did not identify any correlations with hemorrhagic phenomena. This finding is supported by other studies emphasizing that it is not solely the type of anticoagulant therapy but rather the dosage and severity of the SARS-CoV-2 infection that play significant roles in the progression to hemorrhagic phenomena [33,34].

Therapeutic management is categorized into three approaches: surgical intervention, embolization, and conservative therapy. Surgical intervention involves a transabdominal approach for large collections, including evacuation, achieving local hemostasis (when the source is identified), applying hemostatic solutions, and drainage [35]. Moreover, patients with COVID-19 frequently experience a spectrum of clinical complications, including respiratory dysfunction requiring prolonged hospitalization or mechanical ventilation. Beyond hemorrhagic manifestations, this disease is associated with multiple systemic challenges that heighten susceptibility to secondary infections and may ultimately result in severe, potentially fatal outcomes [36].

Surgical solutions are primarily utilized for large collections, imminent risk of intraperitoneal rupture, progression toward hemoperitoneum, severe hemodynamic instability, or when embolization is ineffective. Surgery tends to have a lower success rate compared to the other methods, partly because it is an invasive procedure aimed at patients in critical condition and is often a last resort following the failure of other treatments [37,38].

Regarding the choice between embolization and conservative management, there is a noticeable trend favoring the former, as the results of embolization are superior to those of conservative or surgical management. Additionally, embolization can be used in conjunction with conservative therapy or as a method to evaluate the progress of conservative treatment [39,40].

There is no consensus on the optimal management approach for retroperitoneal hematomas, largely due to the relatively small number of cases with this pathology. The optimal strategy appears to be embolization or conservative management with careful biological, hemodynamic, and imaging monitoring, along with surgical intervention if the evolution is unfavorable [41].

## 4. Conclusions

The occurrence of retroperitoneal hematomas in patients with COVID-19 is attributed to multifactorial mechanisms, including inflammatory–ischemic pathways and the effects of prolonged anticoagulant therapy, rather than being solely due to acute vascular etiologies.

The association between anticoagulant dosing and hemorrhagic complications suggests that both dosage and the severity of SARS-CoV-2 infection are significant determinants in the emergence of bleeding phenomena, rather than the type of anticoagulant administered.

An average interval of 11 days from the initial COVID-19 diagnosis to the detection of retroperitoneal bleeding underscores the necessity for enhanced clinical vigilance and a heightened awareness of nonspecific symptoms in critically ill patients.

Currently, there is no consensus regarding the optimal management of retroperitoneal hematomas, largely due to the paucity of reported cases. The most effective approach appears to be a multimodal strategy that incorporates embolization or conservative management, complemented by diligent monitoring and surgical intervention when clinically indicated.

## Figures and Tables

**Table 1:** Spontaneous retroperitoneal hematomas literature scoping results.

Year	Author	Age	Sex	Debut Day	Symptoms	Location	Dimensions	Anticoagulant Therapy	Hgb	Intervention	Outcome
2021	Yeoh et al., [4]	57	M	10	Back pain	Right	22	Enoxiparin 0.6	5.2	Evacuation, massage	Favorable
2021	Teta et al., [5]	81	F	5	Back pain	Left	25	Lovenox 40 × 2	3.7	Embolization	Exitus
2021	Pálek R et al., [6]	85	F	6	Back pain	Left	18	Nadoparin 0.4 × 2	6.9	Conservative	Exitus
		76	M	6	Right lower limb pain	Right	20	Nadoparin 0.4	5.2	Evacuation, massage	Exitus
2022	Tanal M et al., [7]	47	F	7	Abdominal pain	Left	12	Enoxiparin 0.6	7.7	Conservative	Favorable
		64	M	30	Right lower limb pain	Right	22.5	Enoxiparin 0.6 × 2	14	Embolization	Favorable
2021	Perfecto A et al., [8]	78	M	37	Back pain	Right	17	Enoxaparin 0.6 × 2	14	Embolization	Favorable
		65	F	10	Right lumbar pain	Right	7.8	Enoxiparin 0.4	8.4	Evacuation, massage	Favorable
		57	M	11	Abdominal pain	Left	20	Heparin 5000	6.7	Conservative	Exitus
		87	M	7	Back pain	Right	26	Enoxiparin 0.4 × 2	7.7	Conservative	Exitus
		81	F	9	Back pain	Left	13	Heparin 5000 × 2	9.6	Conservative	Favorable
2020	Sexe J et al., [9]	51	F	10	Back pain	Right	26	Enoxiparin 0.4	8	Conservative	Exitus
2022	Erdinc B et al., [10]	58	F	3	Lower abdominal pain	Left	25	Enoxiparin 0.4 × 2	10.8	Conservative	Exitus
2021	Jalali KS et al., [11]	51	M	14	Back pain	Right	26	Enoxiparin 0.4	7	Conservative	Favorable
		64	M	30	Back pain	Left	22	Enoxiparin 0.4	14	Embolization	Favorable
2023	Evrev D et al., [12]	78	M	37	Back pain	Right	17	Enoxiparin 0.4	14	Embolization	Favorable
		63	F	9	Back pain	Right	12	Enoxiparin 0.4	9.6	Conservative	Favorable
		51	F	2	Back pain	Right	14	Enoxiparin 0.4	7.6	Evacuation, massage	Favorable
		74	M	5	Abdominal pain	Left	18	Enoxiparin 0.4	7.4	Conservative	Favorable
		86	F	10	Abdominal pain	Left	21	Enoxiparin 0.4	4.8	Conservative	Exitus
		80	F	10	Right lumbar pan	Right	20	Enoxiparin 0.4	12	Evacuation, massage	Favorable
		92	F	6	Lower abdominal pain	Right	23	Heparin 5000	6.5	Conservative	Exitus
		86	M	10	Left lower limb pain	Left	14	Enoxiparin 0.6	10	Evacuation, massage	Favorable
		74	M	19	Abdominal pain	Left	23	Enoxiparin 0.6	8.8	Evacuation, massage	Exitus
		65	M	12	Back pain	Right	18	Enoxiparin 0.4	10.8	Conservative	Favorable
		72	M	10	Back pain	Right	21	IV (intravenous)	9	Conservative	Exitus
2022	Dubovský M et al., [13]	74	M	11	Abdominal pain	Left	11	IV (intravenous)	8.8	Evacuation, massage	Favorable
2023	Okada N et al., [14]	49	M	8	Left lower limb pain	Left	17	Clexane 0.8 × 2	9	Evacuation, massage	Exitus
2021	Ohn MH et al., [15]	79	M	9	-	Left	7	Heparin 5000 × 2	8	Embolization	Favorable
		51	F	16	-	Right	16	Enoxiparin 0.6	9.3	Conservative	Exitus
2021	Shah et al., [16]	67	M	2	Back pain	Right	10	Enoxiparin 0.4	13.2	Conservative	Exitus
2022	Hashemi et al., [2]	40	M	4	Back pain	Right	6	Enoxiparin 0.8	9.5	Conservative	Favorable
		70	M	12	Inguinal pain	Left	9	Enoxiparin 0.8	7	Embolization	Favorable
		50	F	7	Back pain	Right	9	Heparin 1000 ui	8.9	Conservative	Favorable
		60	F	10	Back pain	Right	7	Heparin 1000 ui	9.5	Conservative	Favorable
		80	M	3	Back pain	Right	7	Warfarin	9.5	Conservative	Favorable
		70	M	10	Back pain	Right	12	Heparin 7500	8	Embolization	Favorable
2021	Ottewill et al., [17]	88	M	10	Lower abdominal pain	Right	9	Enoxaparin 0.4	-	Conservative	Favorable
		66	M	29	-	Left	13	Enoxiparin 0.8	6.8	Conservative	Exitus
2022	Atanasov et al., [18]	56	F	7	Dysuria	Pelvic	13.9	nadroparin 0.6	7.1	Conservative	Favorable
		67	M	10	Right lumbar pain	Right	12.9	Nadroparin 0.6	7.5	Conservative	Favorable
2020	Patel et al., [19]	69	M	20	Abdominal pain	Right	24	Enoxaparin 0.4	8.4	Embolization	Favorable
2023	Vasković et al., [20]	67	F	15	Abdominal pain	Bilateral	-	Nadroparin 0.6	9.8	Surgery	Favorable
		60	F	16	Abdominal pain	Right	9	Nadroparin 0.6	13	Surgery	Favorable
2020	Scialpi et al., [21]	76	M		NONE	Left	17	Nadroparin 0.6	6.1	Embolization	Exitus
2022	Gupta et al., [22]	63	M	7	Abdominal distension	Left	11	Heparin 0.5	7.2	Embolization	Exitus
		57	F	6	Abdominal pain	Left	10	Heparin 0.5	9	Embolization	Favorable
		63	M	3	Abdominal distension	Right	13	Heparin 0.5	8.9	Conservative	Favorable
2021	Javid et al., [23]	65	M	2	Right hypocondrum pain	Right	12	Heparin 0.5	-	Surgery	Favorable
2021	Mahboubi-Fooladi et al., [24]	65	M	10	Abdominal pain	Left	7.8	Enoxaparin 0.4	8.4	Conservative	Favorable
		57	M	11	Abdominal pain	Right	20	Heparin 0.5	6.7	Conservative	Exitus
		87	M	7	-	Left	-	Enoxiparin 1 g	7.7	Conservative	Exitus
		81	F	9	Abdominal pain	Right	13	Heparin 1000 ui	9.6	Conservative	Favorable
		51	F	16	-	Right	-	Enoxaparin 0.6	8	Conservative	Exitus

M: male; F: female.

**Table 2:** Relationship between symptom locations and their corresponding signs.

Symptom	Right (*n*, %)	Left (*n*, %)	Pelvic (*n*, %)	Bilateral (*n*, %)	Total (*n*, %)
Back pain	17 (31.48%)	4 (7.41%)	0 (0)	0 (0)	21 (38.89%)
Right lower limb pain	2 (3.7%)	0 (0)	0 (0)	0 (0)	2 (3.7%)
Abdominal pain	4 (7.41%)	8 (14.81%)	0 (0)	1 (1.85%)	13 (24.07%)
Right lumbar pain	3 (5.56%)	0 (0)	0 (0)	0 (0)	3 (5.56%)
Lower abdominal pain	2 (3.7%)	1 (1.85%)	0 (0)	0 (0)	3 (5.56%)
Left lower limb pain	0 (0)	3 (5.56%)	0 (0)	0 (0)	3 (5.56%)
Inguinal pain	0 (0)	1 (1.85%)	0 (0)	0 (0)	1 (1.85%)
None	1 (1.85%)	3 (5.56%)	0 (0)	0 (0)	4 (7.41%)
Dysuria	0 (0)	0 (0)	1 (1.85%)	0 (0)	1 (1.85%)
Abdominal distension	1 (1.85%)	1 (1.85%)	0 (0)	0 (0)	2 (3.7%)
Right hypochondrium pain	1 (1.85%)	0 (0)	0 (0)	0 (0)	1 (1.85%)
Total	31 (57.41%)	21 (38.89%)	1 (1.85%)	1 (1.85%)	54 (100%)

**Table 3:** Statistical analysis of symptoms related to hematoma location.

Analyzed Parameters	Statistical Value
Back pain—right retroperitoneum	*p* = 0.029
Abdominal pain—right retroperitoneum	*p* = 0.049
Disuria—right retroperitoneum	*p* = 0.019
Back pain—left retroperitoneum	*p* = 0.029
Abdominal pain—left retroperitoneum	*p* = 0.049
Disuria—right retroperitoneum	*p* = 0.019

**Table 4:** Therapeutic options based on hematoma location.

Intervention	Right (%)	Left (%)	Pelvic (%)	Bilateral (%)	Total (%)
Evacuation	12.96	7.41	0	1.85	22.22
Embolization	9.26	12.96	0	0	22.22
Conservative	35.19	18.52	1.85	0	55.56
Total	57.41	38.89	1.85	1.85	100

**Table 5:** Relationship between Hgb levels and type of therapeutic management.

		%	Minimum	Maximum	Skew	Kurtosis	Mean ± Std.
Hgb	Conservative	55.56%	4.8	15	1.23	2.68	8.6 ± 2.04
	Evacuation	22.22%	5.2	15	0.38	0.09	9.4 ± 2.89
	Embolization	22.22%	3.7	14	0.27	−1.26	9.45 ± 3.61

**Table 6:** Patient outcomes by intervention type.

Intervention	Favorable (%)	Favorable (% Within Outcome)	Exitus (%)	Exitus (% Within Outcome)	Total (%)
Evacuation	16.67	75	5.56	25	22.22
Embolization	16.67	75	5.56	25	22.22
Conservative	29.63	53.33	25.93	46.67	55.56
Total	62.96	-	37.04	-	100

## Data Availability

Not applicable.
